# Benefits of Remote-Based Mindfulness on Physical Symptom Outcomes in Cancer Survivors: Systematic Review and Meta-Analysis

**DOI:** 10.2196/54154

**Published:** 2025-01-16

**Authors:** Maria Komariah, Sidik Maulana, Shakira Amirah, Hesti Platini, Laili Rahayuwati, Ah Yusuf, Mohd Khairul Zul Hasymi Firdaus

**Affiliations:** 1 Faculty of Nursing, Padjadjaran University, Bandung, Indonesia; 2 Faculty of Medicine, University of Indonesia, Depok, Indonesia; 3 Faculty of Nursing, Airlangga University, Surabaya, Indonesia; 4 Kulliyah of Nursing, International Islamic University Malaysia, Selangor, Malaysia

**Keywords:** cancer, physical symptoms, mindfulness, remote-based intervention, quality of life

## Abstract

**Background:**

Many cancer survivors experience a wide range of symptoms closely linked to psychological problems, highlighting the need for psychological treatment, one of the most popular being mindfulness. The use of the internet has greatly increased in the last decade, and has encouraged the use of remote-based interventions to help people living with cancer access treatment remotely via devices.

**Objective:**

The primary aim of this study was to explore the efficacy of internet-based mindfulness interventions on the physical symptoms of people living with cancer, where physical symptoms are defined as distressing somatic experiences (eg fatigue, insomnia, and pain) regardless of the underlying cause. The secondary aim was to investigate interventions for the quality of life (QoL).

**Methods:**

This study followed the Preferred Reporting Items for Systematic Review and Meta-analysis (PRISMA) guidelines. Relevant articles were systematically searched using electronic databases, namely Scopus, Medline through PubMed, Cumulated Index in Nursing and Allied Health Literature (CINAHL) through EBSCOhost, and Cochrane Central Database. Randomized controlled and pilot trials involving adults and/or older adults with cancer and using remote-based mindfulness interventions compared to usual care were included. The quality of the trials included in this study was assessed using the revised Cochrane risk of bias, version 2.0. This study estimated the standardized mean difference (SMD) and mean difference (MD) with 95% CI. The *I*^2^ test was used to identify potential causes of heterogeneity. Publication bias was assessed using contour-enhanced funnel plots and the Egger linear regression test to reveal a small study effect.

**Results:**

The initial search yielded 1985 records, of which 13 studies were ultimately included. After treatment, remote-based mindfulness significantly reduced fatigue (SMD −0.94; 95% CI: −1.56 to −0.33; *P*=.002), sleep disturbance (SMD −0.36; 95% CI: −0.60 to −0.12; *P*=.004), and improved physical function (SMD .25; 95% CI: 0.09 to 0.41; *P*=.002) compared to that observed before treatment. However, compared with usual care, remote-based mindfulness showed a statistically significant reduction only in sleep disturbance (SMD: −0.37; 95% CI: −0.58 to −0.16; *P*=.0006) after treatment. Moreover, remote-based mindfulness was not statistically significant in reducing pain both within and between groups.

**Conclusions:**

Remote-based mindfulness shows promise in reducing sleep disturbances; however, its impact on fatigue, pain, and physical function may be limited.

## Introduction

Advancements in cancer medication have extended the life expectancy of cancer patients in recent years [1]. However, more cancer survivors undergo cancer treatment for a longer period. Chronic treatment has been shown to increase symptom burden and reduce the quality of life (QoL) of cancer survivors [2-7]. More than two-thirds of cancer survivors with advanced disease are symptomatic [8]. Cancer survivors receive supportive care focused on relieving symptoms at all stages of their illness [9-11].

Most cancer survivors frequently experience physical symptoms such as pain and fatigue. Physical symptoms are defined as the subjective experiences of distressing somatic symptoms (eg fatigue, insomnia, pain, and nausea), regardless of the cause [12]. In most cancer survivors, pain may be managed with relatively standard treatment [13]. Recent suggestions include a multimodal approach with tailored therapy, including perceptual, homeostatic, and behavioral reactions to chronic illness. This approach allows healthcare professionals to dynamically manage pain by integrating pharmacological and nonpharmacological strategies (eg, acupuncture and psychotherapy) based on pain pathophysiology and characteristics. Following pain symptoms, 50‐90% of patients experience fatigue, which negatively affects their daily activities and QoL [14]. Insomnia is also a common symptom in cancer survivors and can have a systematic effect on psychological burdens, such as stress, fatigue, and depression [15,16].

The symptoms experienced by cancer survivors and their relationship with psychological problems often benefit from psychotherapy. The benefits of psychotherapy can be explained by the body-mind-spirit model [17], which highlights the interconnectedness of physical, mental, and spiritual health [18]. Commonly used psychotherapies include mindfulness-based stress reduction-based interventions and cognitive behavioral therapy (CBT). These therapies are effective in reducing symptoms in cancer survivors, particularly chronic pain and stress [19-21]. CBT is considered beneficial for alleviating pain and other symptoms by reducing catastrophic thinking and enhancing self-efficacy in coping with symptoms such as pain [22]. Similarly, mindfulness-based interventions are considered beneficial for chronic pain by promoting mindfulness and promoting greater acceptance of pain or other symptoms [22]. Unlike traditional psychotherapies, such as CBT, which primarily focus on cognitive restructuring, mindfulness interventions offer the unique benefit of directly enhancing patients’ capacity for present-moment awareness and acceptance of their experiences.

Advancements in healthcare information technology along with the broader accessibility of healthcare services have driven the rapid growth of remote-based interventions. The intervention spans a wide array of practices and specialties, facilitating interactions through various modalities such as telephone, email, video conferencing, online platforms, and remote monitoring devices. The rapid growth of remote-based methods has led to the delivery of mindfulness through the internet. Remote-based interventions have been integrated into cancer care and treatment, which suggests a benefit in treatment outcomes [23]. Remote-based mindfulness is defined as a psychotherapy program that uses a technological device that ensures interactive and immediate communication and does not require the patient to be present with the therapist [24].

Recent evidence suggests the benefits of remote-based interventions using a website on psychological well-being, such as reducing distress, depression, and anxiety [25-27]. Remote-based interventions may be more suitable for patients who experience weakness and fatigue due to physical limitations, such as cancer survivors. A study conducted by Schellekens et al suggested the benefit of web-based mindfulness-based cognitive therapy programs for improving care outcomes in patients with chronic cancer-related fatigue [28]. While a previous meta-analysis has evaluated the benefit of remote-based mindfulness for cancer survivors [29,30], its focus on physical symptom outcomes remains limited. Therefore, this study aimed to explore the benefit of remote-based mindfulness interventions on physical symptom outcomes as a primary and/or secondary outcome of trial studies in cancer survivors.

## Methods

### Study Design

This study was a systematic review and meta-analysis. This study was presented in accordance with the preferred reporting items for systematic reviews and meta-analyses (PRISMA; Checklist 1) [31]. The protocol was not prospectively registered in any database such as PROSPERO (Prospective Register of Systematic Reviews).

### Eligibility Criteria

The inclusion criteria were defined according to the Population, Intervention, Comparison, Outcome (PICO) framework. The population of the included studies was diagnosed with cancer through imaging, laboratory tests (including tumor marker tests), tumor biopsies, endoscopic examinations, surgeries, and genetic testing. Interventions were remote-based mindfulness interventions defined as mindfulness interventions that integrated information and communication technology, such as mobile phones, websites, mobile apps, and asynchronous instruction with text-based reminder messages. Comparisons were defined as standard or usual care with face-to-face mindfulness interventions, or standard cancer care. The outcomes of this study included the physical symptoms related to cancer outcomes. Physical symptoms were defined as the subjective experiences of distressing somatic symptoms (eg, fatigue, insomnia, and pain). The outcomes were measured using self-reports or standard questionnaires. The exclusion criteria were the types of articles, such as case reports, editorials, invited commentary, reviews, non-research letters, and abstract-only articles. To prevent bias, articles published before 2012 and those written in a language other than English as an international language were excluded from this study. This review focused on studies published after 2012 to ensure that the findings represented the most recent advancements in technology, healthcare practices, and guidelines that have progressed markedly over the past decade.

### Study Search Strategy and Selection Process

The selection process for this study followed the Preferred Reporting Items for Systematic Reviews and Meta-Analyses protocol. This review systematically searched electronic databases, namely Scopus, Medline, PubMed, Cumulated Index in Nursing and Allied Health Literature (CINAHL), EBSCOhost, and the Cochrane Central Database. The search was conducted until December 2022. The following keywords were used. (All Fields] OR Internet-based intervention “web-based”[All Fields] OR “internet-based intervention”[All Fields] OR “online based”[All Fields]) AND (“mind s”[All Fields] OR “minded”[All Fields ] OR “mindful”[All Fields] OR “mindfulness”[MeSH Terms] OR “mindfulness”[All Fields] OR “mindfulness intervention”[All Fields] OR “mindfulness-based stress reduction”[All Fields] OR “mindfulness- based cognitive therapy”[All Fields]) AND (“cancer s”[All Fields] OR “cancer”[All Fields] OR “cancers”[All Fields] OR “oncology patients”[All Fields] OR “Patients with cancer” [All Fields]). The detailed search strategy can be found in Multimedia Appendix 1. In addition, we used a hand-searched reference list of the included studies to expand the number of additional studies.

The reference manager automatically removed duplicate articles using Mendeley (Mendeley Ltd.). Two independent authors (SM and SA) initially screened the text (eg, title and abstract). The full text of the articles that met the eligibility criteria were independently assessed by two independent authors. At this stage, the articles were meticulously evaluated based on predetermined inclusion and exclusion criteria, and irrelevant studies were excluded. Discrepancies were resolved by a third reviewer (MK).

### Data Extraction

Two authors (MK and SM) independently extracted data using standard tabulation tables (spreadsheets). The following data were included: study characteristics (ie, author, year, study design, country, model intervention, and follow-up duration); participant characteristics (ie, average age, education level, number of participants, and cancer site); and physical symptoms (eg pain, fatigue, and insomnia). Data extraction was performed independently and disagreements were resolved through discussion and consensus among the authors.

This study assessed the quality of this randomized-controlled trial (RCT) using the Cochrane risk of bias, version 2.0. Three authors (MK, SM, and HP) evaluated the enrolled studies separately. The following factors were considered in the assessment: bias arising from random processes, bias due to deviation from the intended intervention, bias due to missing outcome data, bias in outcome measures, and bias in selection of reported outcomes. This discourse resolved the differences in perceptions regarding the quality of the research.

### Statistical Analysis

All statistical analyses were performed using Review Manager version 5.4.1 (RevMan) [32]. This study estimated the effect size in the form of the standardized mean difference (SMD) for the outcome and the mean difference (MD), with the 95% CI. The SMD was used when the outcomes were measured in different units across studies. The MD was used when the outcomes were measured in the same unit across studies. The SMD criteria were divided into three categories: low, medium, and large effects, with values of <0.5, ≥05, and ≥0.8, respectively [33]. This review conducted posttreatment analysis that reported pre- and post–remote-based intervention. We also conducted a comparison between remote-based intervention and usual care after treatment. The inconsistency index (*I*^2^) and subgroup analysis using the *I*^2^ test were used to identify potential causes of heterogeneity. An *I*^2^ value of >50% and a *P*-value of <.05 were considered statistically significant for heterogeneity [34]. A random-effects model was applied despite the study heterogeneity to account for interstudy variability [35]. In this study, a two-tailed *P *value of .05 was considered statistically significant. Publication bias was analyzed qualitatively using a contour-enhanced funnel plot and quantitatively using the Egger linear regression test.

## Results

### Study Selection

**Figure 1.**
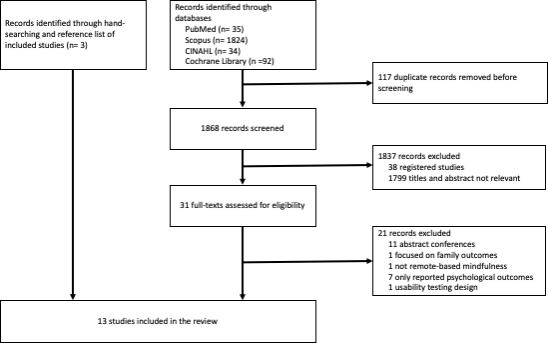
Study selection process.

The process of selecting the studies for inclusion in the review is presented in Figure 1. An initial search across PubMed, CINAHL, Scopus, and Cochrane Library databases yielded 1985 articles. A total of 177 duplicate articles were removed before screening, resulting in 1868 articles. After screening, 1837 studies were excluded because of 38 preregistered studies (eg ClinicalTrials.gov), and 1799 titles and abstracts were not relevant. After assessing 31 full-text articles for eligibility, 21 studies were conference abstracts, focused on family outcomes, not remote-based mindfulness or usability testing, and did not report the physical outcomes. Ten studies met the criteria identified through the database, and 3 studies were identified through manual searches and reference lists of the included studies. Hence, 13 studies were included in the systematic review and meta-analysis [26,36-47].

### Characteristics of the Included Studies

The mean age of the participants was <60 years in 10 of the included studies and ≥60 years in 2 studies. Most participants were female, with 74.38% (572/894) in the remote-based intervention group and 70.61% (322/894) in the usual care group. The studies were conducted across several countries, with most studies conducted in the United States (n=4) and the Netherlands (n=4), followed by China (n=2), and 1 each in Ireland, Denmark, and Iran. Regarding the study design, 11 studies were RCTs and 2 were pilot RCTs. The mindfulness type included web-based interventions, mobile apps, and virtual meetings, whereas the control groups included treatment as usual, wait-list controls, face-to-face mindfulness, and interventions without a control group. The outcome measurements included assessments of fatigue, sleep disturbances, and physical function. Fatigue was measured in 5 studies by using different instruments, such as the checklist of individual strength (CIS)-fatigue, BFI-9, fatigue symptom inventory, and cancer quality of life questionnaire (QLQ)-C30. Sleep disturbance was evaluated in 6 studies using tools such as the patient-reported outcomes measurement information system (PROMIS), Pittsburgh sleep quality index (PSQI), and insomnia severity index (ISI). Physical function was measured in 7 studies, most frequently using the short form (SF)-12, functional assessment of chronic illness therapy (FACIT), and QLQ-C30. The details of these characteristics are presented in Table 1 and Multimedia Appendix 2.

**Table 1.** Characteristic of the included studies (n=13).

Characteristics		
Mean age (years)	Number of studies (n=13)	Reference
<60	10	[36-41,43-45,47]
≥60	2	[26,42]
Data not available	1	[46]
Sex (Female)	n (%)	Reference
Remote-based group	572 (74.38)	NA
Usual care	322 (70.61)	NA
Country	Number of studies (n=13)	Reference
United States of America	4	[41,42,44,45]
Ireland	1	[40]
Netherlands	4	[36-39]
Denmark	1	[26]
China	2	[43,46]
Iran	1	[47]
Study design		Reference
RCT^a^	11	[26,37-43,45-47]
Pilot-RCT	2	[36,44]
Type of mindfulness delivered	Number of studies (n=13)	Reference
Web-based	5	[36-39,43]
Mobile apps	4	[26,41,42,44]
Virtual meeting	2	[46,47]
Unspecified	2	[40,45]
Type of control group	Number of studies (n=13)	Reference
Treatment as usual	6	[38-40,45-47]
Wait-list control	4	[26,41-43]
Face-to-face mindfulness	1	[37]
Without control	2	[36,44]
Fatigue measurement	Number of studies (n=5)	Reference
CIS-Fatigue^b^	1	[36]
BFI-9	1	[41]
FSI^c^	2	[44,45]
QLQ-30^d^	1	[47]
Sleep disturbance measurement	Number of studies (n=6)	Reference
PROMIS^e^	1	[41]
PSQI^f^	3	[43-45]
ISI^g^	2	[26,47]
Physical function measurement	Number of studies (n=7)	Reference
SF-12^h^	4	[37-39,44]
FACIT^i^	2	[41,42]
QLQ-30	1	[46]

a RCT: randomized-controlled trial.

b CIS-fatigue: checklist individual strength for fatigue.

c FSI: fatigue symptom inventory.

d QLQ-C30: Cancer Quality of Life Questionnaire- C30.

e PROMIS: patient-reported outcome measurement information system.

f PSQI: Pittsburgh Sleep Quality Index.

g ISI: insomnia severity index.

h SF-12: short form-12 items.

i FACIT: Functional Assessment of Chronic Illness Therapy.

### Study Outcomes

A meta-analysis of remote-based mindfulness revealed 4 physical outcomes. The outcomes included fatigue (n=5), sleep disturbance (n=6), pain (n=3), and physical function (n=6). The outcome measurements varied, as shown in Table 1. The effect sizes for each outcome are listed in Table 2.

**Table 2.** Effect size of mobile-based mindfulness on physical symptoms in cancer survivors.

Outcome	Number of studies	Effect size	95% CI	*P* value	Heterogeneity	Reference
Pre- and postintervention						
Fatigue	5	SMD^a^ −0.94	−1.56 to −0.33	.002*^b^	85%	[36,41,44,45,47]
Sleep disturbance	6	SMD −0.36	−0.60 to −0.12	.004*	31%	[26,41,43-45,47]
Pain	3	MD^c^ −5.33	−10.90 to 0.25	.06	85%	[40,41,44]
Physical function	6	SMD 0.25	0.09 to 0.41	.002*	0%	[37-39,41,44,46]
Controlled intervention						
Fatigue	3	SMD −1.09	−2.87 to 0.68	.23	95%	[41,45,47]
Sleep disturbance	5	SMD −0.37	−0.58 to −0.16	.006*	46%	[26,41,43,45,47]
Pain	2	MD −0.90	−2.31 to 0.52	.21	0%	[40,41]
Physical function	5	SMD 0.59	−0.06 to 1.24	.08	92%	[38,39,41,42,46]

a SMD: Standard mean difference.

b The asterisk indicates statistical significance (*P*<.05)

c MD: Mean difference.

### Pre- and Postanalysis of Remote-Based Mindfulness to Physical Outcomes After Treatment

**Figure 2.**
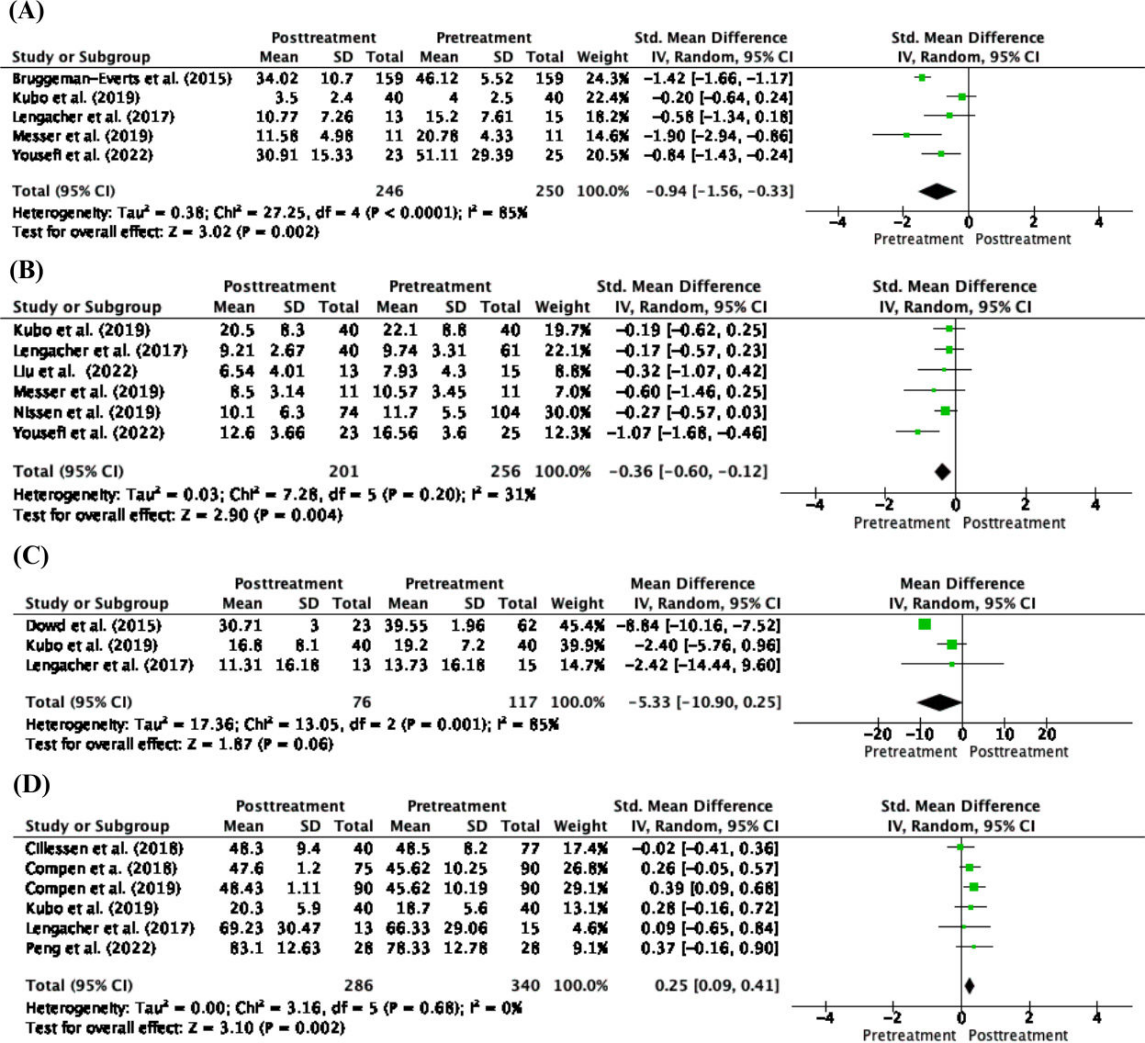
Meta-analysis of the benefits of remoted-based mindfulness intervention on physical symptoms after treatment. (**A**) Fatigue outcome. (**B**) Sleep disturbance outcome. (**C**) Pain outcome. (**D**) Physical function.

After remote-based mindfulness treatment, cancer survivors showed a significant reduction in fatigue (SMD −0.94; 95% CI: −1.56 to −0.33; *P*=.002), sleep disturbance (SMD −0.36; 95% CI: −0.60 to −0.12; *P*=.004), and improvement in physical function (SMD 0.25; 95% CI: 0.009 to 0.41; *P*=.002) compared with baseline or pretreatment values. Although posttreatment outcomes were more favorable compared to baseline values, there was no statistically significant difference in pain reduction (MD −5.33; 95% CI: −10.90 to 0.25; *P*=.06; Table 2). A forest plot of the pre- and posttreatment meta-analyses conducted on the remote-based mindfulness group is shown in Figure 2. Among these 4 outcomes, fatigue and pain showed significant heterogeneity (*I*^2^=85%).

### Benefits of Remote-Based Mindfulness on Physical Symptoms Compared to Usual Care After Treatment

**Figure 3.**
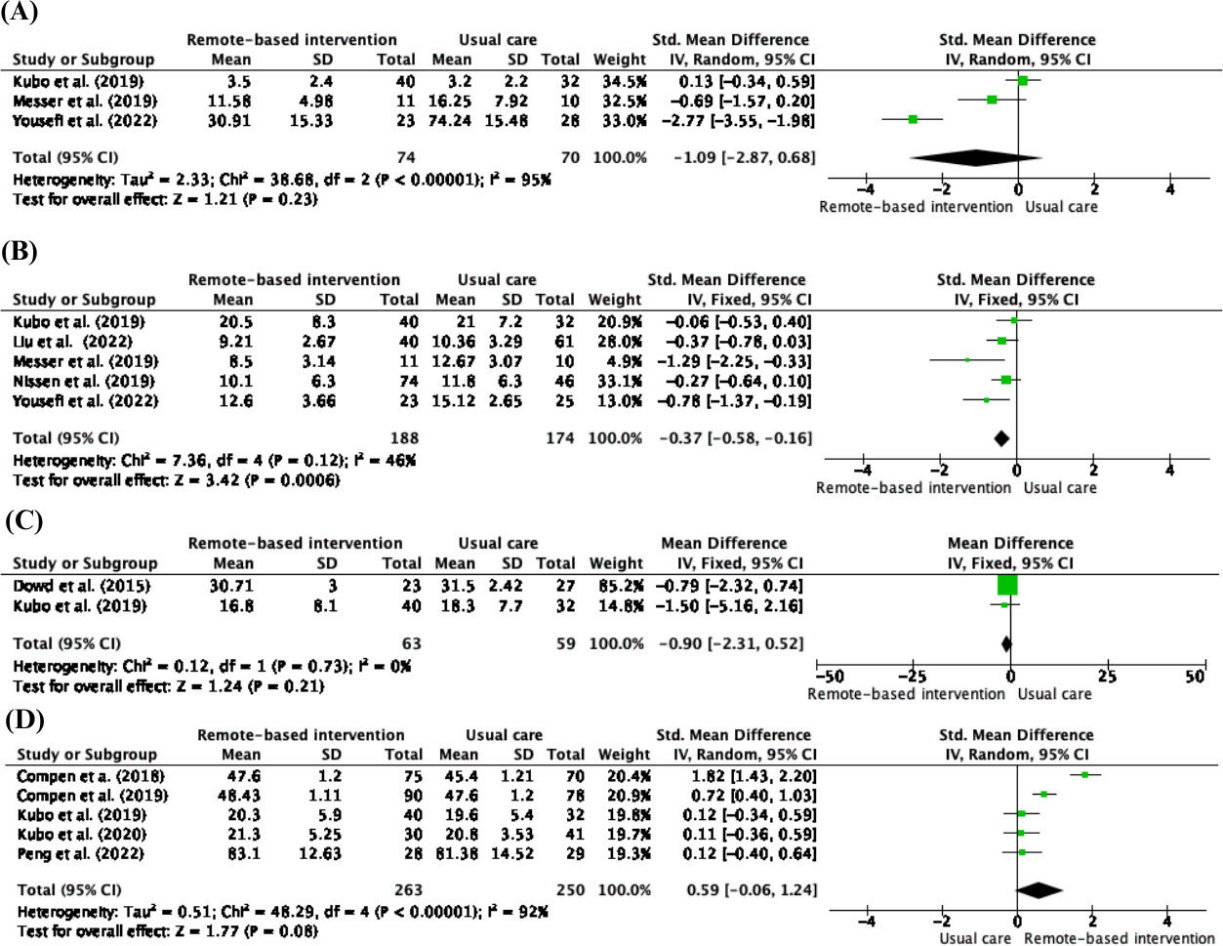
Meta-analysis of the benefits of remote-based mindfulness intervention on physical symptoms compared to usual care. (**A**) Fatigue outcome. (**B**) Sleep disturbance outcome. (**C**) Pain outcome. (**D**) Physical function.

Despite the small effect, the meta-analysis showed that remote-based mindfulness significantly reduced sleep disturbance (SMD −0.37; 95% CI: −0.58 to −0.16, *P*=.0006) compared with usual care after treatment. There were no statistically significant differences in the reduction of fatigue, pain, or improvement of physical function between the remote-based mindfulness and usual care groups (Table 2). Although not statistically significant, the remote-based mindfulness group had reduced fatigue, sleep disturbance, and pain compared with the usual care group after treatment. The forest plot of the meta-analysis of the benefits of remote-based mindfulness compared to usual care after treatment is shown in Figure 3.

### Quality Assessment

**Figure 4.**
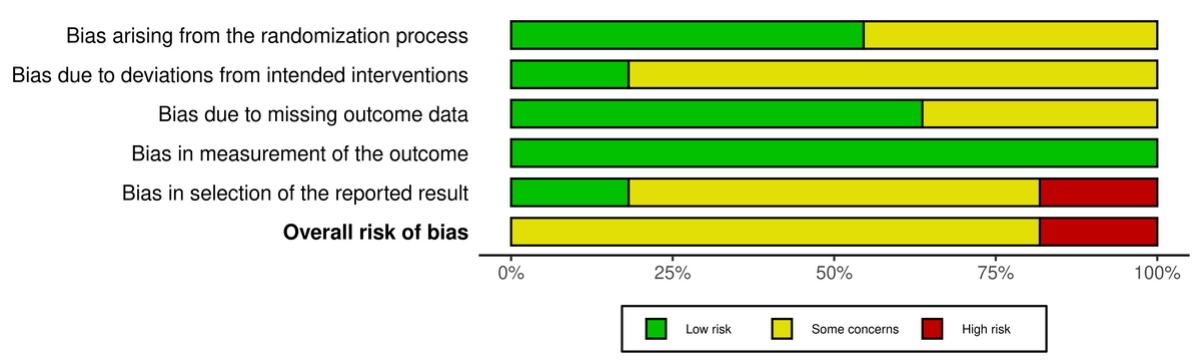
Summary risk of bias.

Over 75% of the studies showed some concerns in at least 1 domain, but no study was rated as high risk considering the measurement of the outcomes (Figure 4). Most studies showed a low risk of bias across most domains, particularly for bias in the measurement of outcomes and missing outcome data. However, some concerns were found regarding the bias arising from the randomization process and deviations from intended interventions, with several studies lacking sufficient details on allocation concealment or participant adherence. Two studies, notably those by Cillessen et al (2018) and Nissen et al (2019), demonstrated a high risk of bias in the selection of the reported results. These studies may have selectively reported favorable outcomes, raising concerns about the validity of their findings. A detailed assessment of each included study can be found in the traffic-light plot provided in Multimedia Appendix 3.

### Publication Bias

We evaluated the likelihood of publication bias by analyzing funnel plots and using the Egger test. We did not conduct statistical tests or create funnel plots for any outcome because each outcome had fewer than 10 studies, which is necessary to ensure sufficient power for detecting asymmetry [29,48].

## Discussion

### Study Findings and Comparison With Previous Works

To the best of our knowledge, this systematic review and meta-analysis is the first to assess the benefits of remote-based mindfulness interventions on physical outcomes in individuals living with cancer. This study has yielded several findings. First, the meta-analysis concluded that a significant effect was observed in reducing fatigue and sleep disturbance after treatment. Second, remote-based mindfulness was significantly more effective in reducing sleep disturbances compared to usual care. Third, remote-based mindfulness was not significantly effective at reducing pain. Finally, a significant improvement in physical function was observed after treatment.

The present meta-analysis suggests that remote-based mindfulness is beneficial for improving physical outcomes. The present study adds to the knowledge regarding the benefits of remote-based mindfulness in cancer survivors. A previous meta-analysis suggested that remote-based mindfulness reduces psychological symptoms in cancer survivors, such as depression, distress, and perceived stress [30,49]. Another meta-analysis observed a significant effect of remote-based mindfulness with a specific web-based platform in reducing anxiety, depression, and distress [29].

The biological mechanisms underlying the benefits of mindfulness treatments suggest additional pathways that may strengthen evidence-based understanding of their physical health effects. Preliminary supporting studies indicate that mindfulness interventions promote two pathways of stress resilience in the brain (the regulatory and reactivity pathways) and may enhance the regulation of the stress reactivity of the hypothalamic-pituitary-adrenal and sympathetic-adrenal-medullary axes, thereby elucidating the effects of mindfulness interventions on stress-related health and disease outcomes over time [50]. The effectiveness of remote-based mindfulness can be understood through the body-mind-spirit model, in which physical health is influenced by the interconnectedness of biological and psychological factors involving self-regulation [17,18]. This self-regulation encompasses the release of dopamine, endocannabinoids, endorphins, and stress hormones in addition to the signaling pathways of oxytocin and serotonin [51].

The present meta-analysis showed a significant effect in reducing sleep disturbance compared with usual care, which is consistent with the findings of a previous meta-analysis [29]. Mindfulness treatment has the potential to alleviate sleep disturbances because mindfulness practice enables individuals to observe their thoughts, emotions, and bodily sensations without emotional involvement or judgment [52]. It also seeks to increase an individual’s awareness and acceptance of their thoughts, emotions, and physiological sensations. This treatment improves cognitive flexibility and cultivates a more comprehensive understanding of sleep, thereby alleviating anxiety or arousal, which may exacerbate sleep disturbances [30].

Despite the present meta-analysis showing that remote-based mindfulness significantly reduced fatigue after treatment, the results showed no significant difference when compared with usual care. Consistent with a previous meta-analysis of web-based mindfulness, there was no significant effect compared to usual care [30]. This may align with the different types of cancer and stages, types of technological intervention, treatment duration, and diverse measurement instruments within the studied population. Despite this, remote-based mindfulness showed high effectiveness after treatment, which aligns with a previous meta-analysis of face-to-face mindfulness [53]. A meta-analysis conducted by Johns et al showed a moderate effect after treatment and a small effect at the first-month follow-up [53]. Remote-based mindfulness is well-documented for its efficacy in reducing and managing stress, which may subsequently impact fatigue. Furthermore, fatigue may be alleviated by enhancing insomnia, as better sleep quality leads to increased freshness [47]. Peripheral inflammatory cytokines can communicate with the central nervous system to induce cancer-related fatigue [54]. Mindfulness, such as the body-mind-spirit technique, may reduce NF-kB signaling, a major regulator of inflammatory activity [55].

This meta-analysis showed no significant difference in pain reduction compared to usual care. This outcome may be attributed to the fact that both the remote-based and control groups were provided with standard care, which included adequate analgesic administration as part of their standard treatment protocol [56]. Mindfulness-based interventions may have been marked by the high efficacy of analgesics in alleviating chronic pain in cancer survivors. A previous meta-analysis of face-to-face mindfulness showed only a small effect in reducing chronic pain in various health conditions [57]. A psychotherapy form similar to online-based acceptance and commitment therapy showed moderately reduced chronic pain in various health conditions [58].

Evidence suggests that remote-based mindfulness improves QoL [29], with no exception to the present meta-analysis, which showed that remote-based mindfulness significantly improved the physical function of QoL after treatment. By reducing cancer-related symptoms, including physical symptoms, remote-based mindfulness can improve physical function. However, the present meta-analysis concluded that there was no significant improvement in physical function compared with usual care. This result may largely benefit psychological outcomes rather than physical health outcomes.

### Future Direction

This evidence suggests a potential remote-based mindfulness intervention to alleviate physical symptoms (eg, sleep disturbance and fatigue) and improved physical function. The understanding of mindfulness interventions, including remote-based mindfulness, and their benefit on physical health remains insufficient considering the large RCT literature associating mindfulness interventions with psychological outcomes [50,59]. Further research is needed to evaluate the efficacy of remote-based mindfulness in improving physical outcomes (eg, blood pressure, weight loss, and biomarkers of health). Integrating mindfulness practices into supportive care programs acknowledges the importance of addressing multidimensional aspects of a patient’s experience. This personalized and holistic approach aligns with the principles of patient-centered care, recognizing the unique needs and challenges faced by individuals undergoing cancer treatment.

Despite the small number of included studies, the evidence of the pain outcomes suggests the limited benefit of remote-based mindfulness intervention due to the administration of standard analgesics in both groups [56]. Considering the analgesic effects induced within the central nervous system, the common adverse effects of opioids include nausea, vomiting, constipation, drowsiness, disorientation, hallucinations, and respiratory depression. Other adverse effects include endocrine alterations (eg, androgen insufficiency and bone demineralization) and the risk of depression due to long-term opioid prescriptions [51]. Owing to the growing “opioid crisis,” the use of opioids as a psychotherapy option is now being recommended as a complementary treatment. Hence, further research and modification of mindfulness interventions with other psychotherapies is needed to enhance the benefits and evidence of remote-based mindfulness on pain.

### Limitations

Despite this present study indicating the potential effects of remote-based mindfulness on physical health outcomes and physical status, our study has several limitations. This meta-analysis was not registered prospectively in any registered database such as PROSPERO. The transparency of this meta-analysis was limited because of the minimized risk of selective reporting. A few studies included in the meta-analysis had a high bias in the selection of the reported results that influenced the concern that positive results are more likely to be published. Meta-regression was not performed in the present meta-analysis to assess potential moderating factors such as participant characteristics, intervention components, or variations in study design. Moreover, this systematic review and meta-analysis assessed mindfulness as psychotherapy, and the included studies were unlikely to evaluate physical health outcomes as primary outcomes.

### Conclusion

This meta-analysis provided evidence regarding remote-based mindfulness interventions to alleviate physical symptoms in cancer survivors. The findings of this study suggest that remote-based mindfulness interventions may be effective in reducing sleep disturbances in clinical practice. Despite limited evidence regarding its benefits compared with usual care, the effect of remote-based mindfulness on fatigue and physical function was observed after treatment. Due to the limited number of included studies and the heterogeneity of the included studies, the conclusions must be considered along with these limitations. Therefore, well-designed trials are required to obtain robust evidence.

## Supplementary material

10.2196/54154 Multimedia Appendix 1 Search strategy.

10.2196/54154 Multimedia Appendix 2 Characteristic of the included studies.

10.2196/54154 Multimedia Appendix 3 Traffic light plot.

10.2196/54154 Checklist 1 PRISMA Checklist.

## Abbreviations

CBT: cognitive behavioral therapy
CINAHL: Cumulated Index in Nursing and Allied Health Literature
FACIT: functional assessment of chronic illness therapy
ISI: insomnia severity index
MD: mean difference
PICO: Population, Intervention, Comparison, Outcome
PROMIS: patient-reported outcomes measurement information system
PROSPERO: Prospective Register of Systematic Reviews
PSQI: Pittsburgh sleep quality index
QLQ: quality of life questionnaire
QoL: quality of life
RCT: randomized-controlled trial
SF: short form
SMD: standardized mean difference
