# Correction: Benefits of Remote-Based Mindfulness on Physical Symptom Outcomes in Cancer Survivors: Systematic Review and Meta-Analysis

**DOI:** 10.2196/71958

**Published:** 2025-02-13

**Authors:** Maria Komariah, Sidik Maulana, Shakira Amirah, Hesti Platini, Laili Rahayuwati, Ah Yusuf, Mohd Khairul Zul Hasymi Firdaus

**Affiliations:** 1 Faculty of Nursing, Padjadjaran University, Jl. Ir. Soekarno KM. 21, Jatinangor, Bandung, 45363, Indonesia, 62 81294686288; 2 Faculty of Medicine, University of Indonesia, Depok, Indonesia; 3 Faculty of Nursing, Airlangga University, Surabaya, Indonesia; 4 Kulliyah of Nursing, International Islamic University Malaysia, Selangor, Malaysia

In “Benefits of Remote-Based Mindfulness on Physical Symptom Outcomes in Cancer Survivors: Systematic Review and Meta-Analysis” (JMIR Cancer 2025;11:e54154) an error was noted.

Reference 46 was previously a duplicate of reference 26, as follows:

*Nissen ER, O’Connor M, Kaldo V, et al. Internet-delivered mindfulness-based cognitive therapy for anxiety and depression in cancer survivors: A randomized controlled trial. Psychooncol. Jan 2020;29(1):68-75*.

All in-text citations to reference 46 have been changed to 26, the repeated reference information removed, and all subsequent references renumbered accordingly.

The correction will appear in the online version of the paper on the JMIR Publications website on February 13, 2025, together with the publication of this correction notice. Because this was made after submission to PubMed.

