# Supplementary material for: Benefits of Remote-Based Mindfulness on Physical Symptom Outcomes in Cancer Survivors: Systematic Review and Meta-Analysis
Source: JMIR Cancer. 2025 Jan 16;11:e54154. doi: 10.2196/54154 (PMC11870029; doi:10.2196/54154)
Supplement: Multimedia Appendix 1 [file cancer-v11-e54154-s001.docx]

**Multimedia Appendix 1.**

| Database | Keyword | Retrieved | Date |
| --- | --- | --- | --- |
| PubMed | ("web-based"[All Fields] OR "internet-based intervention"[All Fields] OR "online based"[All Fields]) AND ("mind s"[All Fields] OR "minded"[All Fields ] OR "mindful"[All Fields] OR "mindfulness"[MeSH Terms] OR "mindfulness"[All Fields] OR “mindfulness intervention”[All Fields] OR “mindfulness-based stress reduction”[All Fields] OR “mindfulness- based cognitive therapy”[All Fields]) AND ("cancer s"[All Fields] OR "cancer"[All Fields] OR "cancers"[All Fields] OR “oncology patients”[All Fields] OR “Patients with cancer” [All Fields]). | 35 | December 30, 2023 |
| Scopus | web-based* OR "web-based intervention" OR internet-based* OR "internet-based intervention" OR "online intervention" AND mindfulness OR "mindfulness-based stress reduction" OR "mindfulness-based cognitive therapy" AND cancer OR "oncology patients" OR "patients with cancer". | 1824 | December 30, 2023 |
| CINAHL | ( web-based interventions' or 'e-health' or 'internet-based interventions or internet-based or digital or online intervention ) AND ( mindfulness based stress reduction or mindfulness or mbsr or mindfulness intervention ) AND ( cancer patients or oncology patients or patients with cancer ). | 34 | December 30, 2023 |
| Cochrane | web-based* OR "web-based intervention" OR internet-based* OR "internet-based intervention" OR "online intervention" AND mindfulness OR "mindfulness-based stress reduction" OR "mindfulness-based cognitive therapy" AND cancer OR "oncology patients" OR "patients with cancer". | 92 | December 30, 2023 |
