# Supplementary material for: Benefits of Remote-Based Mindfulness on Physical Symptom Outcomes in Cancer Survivors: Systematic Review and Meta-Analysis
Source: JMIR Cancer. 2025 Jan 16;11:e54154. doi: 10.2196/54154 (PMC11870029; doi:10.2196/54154)
Supplement: Multimedia Appendix 2 [file cancer-v11-e54154-s002.docx]

**Multimedia Appendix 2. Characteristic of included studies**

| **No** | **Study** | **Location** | **Design** | **Control group** | **Intervention group** | **Population** | **Age (N, SD)** | **Gender (Female %)** | **Number of female** | **Follow-up** | **Outcome** | **Instrument** | **Type of outcome measurement** |
| --- | --- | --- | --- | --- | --- | --- | --- | --- | --- | --- | --- | --- | --- |
| 1 | Bruggeman-Everts et al. (2015) | Netherlands | A Pilot RCT | NA | Audio Web-page that contained mindfulness material and asynchronous interaction via email | Cancer patients | 50.2 (10.7) | 76 | 196/257 | 9 weeks | Fatigue | CIS-Fatigue | Primary |
| 2 | Cillessen et al. (2018) | Netherlands | RCT | Face to face mindfulness | Web-page that contained mindfulness material and asynchronous interaction via email | Distress cancer patient | IG: 51.5 (11.1) CG: 51.8 (10.2) | IG: 87.2 CG: 84.2 | IG: 109/125 CG: 101/120 | 8 weeks | Physical function | SF-12 | Secondary |
| 3 | Compen et a. (2018) | Netherlands | RCT | Treatment as usual | Web-page that contained mindfulness material and asynchronous interaction via email | cancer patients | IG: 52.4 (10.7) CG: 50.4 (9.9) | IG: 85.6 CG: 84.6 | IG: 77/90 CG: 66/78 | 5 months | Physical function | SF-12 | Secondary |
| 4 | Compen et al 2019 | Netherlands | RCT | Treatment as usual | Web-page that contained mindfulness material and asynchronous interaction via email | cancer patients | IG: 52,4 (10,7) CG: 52.4 (9.9) | IG : 85.6 CG: 84.6 | IG: 77/90 CG: 66/78 | 8 weeks | Physical function | SF-12 | Secondary |
| 5 | Dowd et al. (2015) | Ireland | RCT | Treatment as usual | Guided meditation audio and textual lessons | cancer patients | 44.53 (12.25) | 90.3 | IG+CG= 112/124 | 6 weeks | Pain | Pain interference | Primary |
| 7 | Kubo et al. (2019) | USA | RCT | Wait-list control | Commercial website accessed mobile apps and asynchronous interaction via email | Cancer Patients | IG: 59.3 (14.1) CG: 56.7 (14.7) | IG: 62.3 CG: 76.7 | IG: 33/54 CG: 33/43 | 8 weeks | Fatigue Sleep disturbance Pain Physical function | BFI-9 PROMIS Pain interference FACIT | Primary |
| 6 | Kubo et al. (2020) | USA | RCT | Wait-list control | Commercial website accessed mobile apps and asynchronous interaction via email | Advanced cancer patients | IG: 65.8 (8.8) CG: 67.1 (10.4) | IG: 73.1 CG: 66.7 | IG: 38/52 CG: 34/51 | 12 weeks | Physical function | FACIT | Primary |
| 8 | Liu et al 2022 | China | RCT | Wait-list control | Guided meditation audio accessed via web and asynchronous interaction via WeChat | Patients with hepatocellular carcinoma | IG: 54.36 (8.46) CG: 57.02 (8.00) | IG: 24.6 CG: 21.3 | IG: 15/61 CG: 13/61 | 6 weeks | Sleep disturbance | PSQI | Secondary |
| 9 | Lengacher et al 2018 | USA | A Pilot RCT | NA | Guided meditation audio and psychoeducation modules accessed mobile apps | breast cancer patients | 57 (9) | 100 | 15/15 | 6 weeks | Fatigue Sleep disturbance Pain Physical function | FSI PSQI Pain interference SF-12 | Primary |
| 10 | Messer et al. (2019) | USA | RCT | Treatment as usual | Guided meditation audio and textual lessons | Cancer diagnosis stage I–III with treatment complete | 51 | 76 | 16/21 | 6 weeks | Fatugue Sleep disturbance | FSI PSQI | Primary |
| 11 | Nissen et al 2019 | Denmark | RCT | Wait-list control | Guided meditation audio accessed mobile phone and asynchronous interaction | Patient with cancer | Breast cancer= 54.55 Prostate cancer= 64.94 | NA | NA | 6 months | Sleep disturbance | ISI | Secondary |
| 12 | Peng et al. (2022) | China | RCT | Treatment as usual | Online group structured mindfulness | Brest cancer patients | NA | 100 | 57/57 | 6 weeks | Physical function | QLQ-30 | Primary |
| 13 | Yousefi et al. (2022) | Iran | RCT | Treatment as usual | Online group structured mindfulness | Patient with colorectal and stomach cancers | IG: 53.16 (7.35) CG: 56.64 (5.46) | IG: 48 CG: 36 | IG: 12/25 CG: 9/25 | 9 weeks | Fatigue Sleep disturbance | QLQ-30 Fatigue ISI | Primary |
