# Supplementary figures and images for: Benefits of Remote-Based Mindfulness on Physical Symptom Outcomes in Cancer Survivors: Systematic Review and Meta-Analysis
Source: JMIR Cancer. 2025 Jan 16;11:e54154. doi: 10.2196/54154 (PMC11870029; doi:10.2196/54154)

**Multimedia Appendix 3. Traffic light plot**


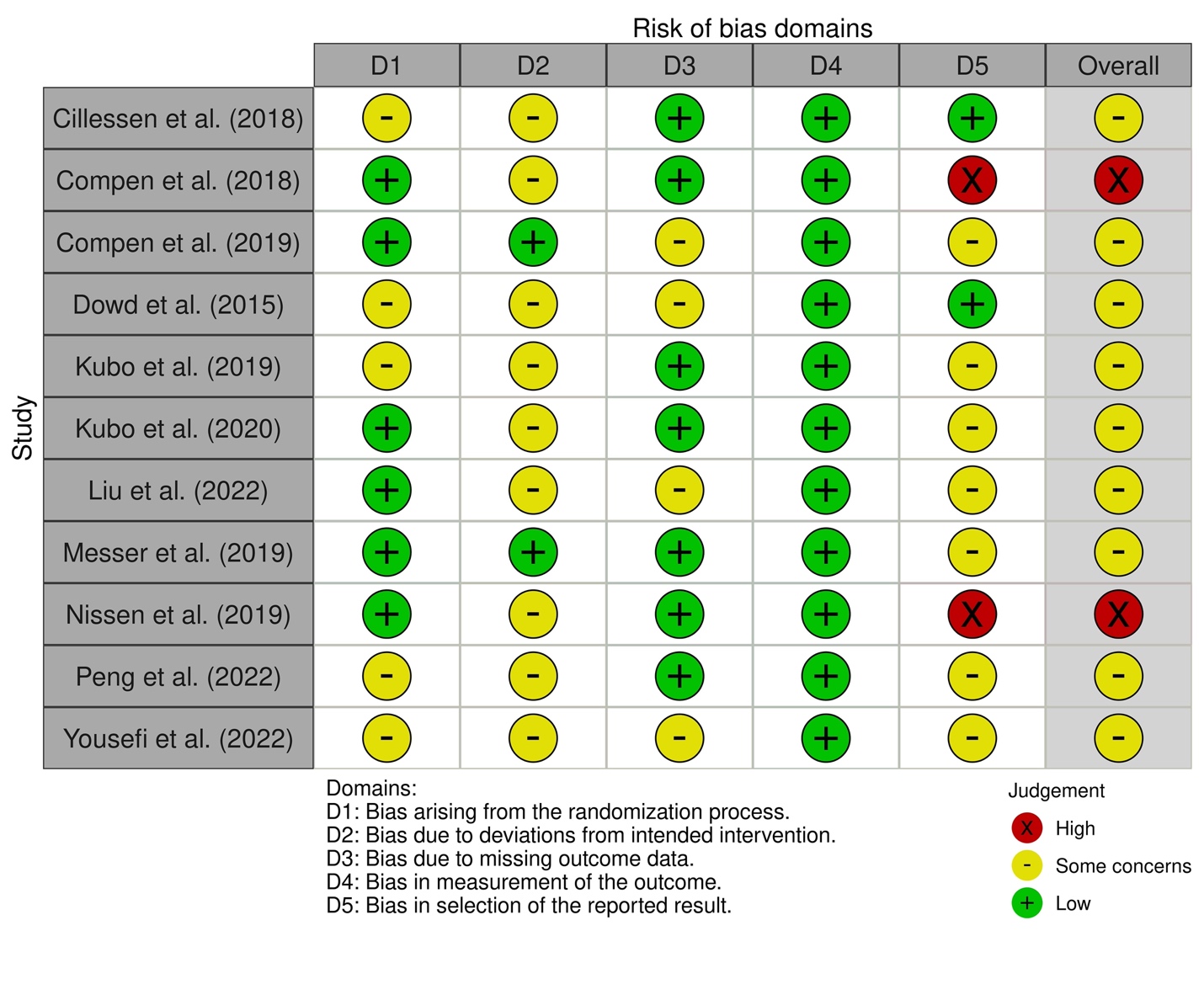

Supplement: Multimedia Appendix 3 [file cancer-v11-e54154-s003.docx]
